# P-10. Clinical Outcomes of Standard-of-Care versus Broad-Spectrum Beta-Lactams for Methicillin-Susceptible Staphylococcus aureus Bacteremia

**DOI:** 10.1093/ofid/ofaf695.241

**Published:** 2026-01-11

**Authors:** Catherine Smith, Emerald O’Rourke, Elizabeth Arnold, Michelle Lee

**Affiliations:** Brown University Health Rhode Island Hospital, Stow, MA; Brown University Health, Newport, RI; University of Rhode Island College of Pharmacy, Kingston, Rhode Island; Brown University Health Rhode Island Hospital, Stow, MA

## Abstract

**Background:**

The standard-of-care (SOC) antibiotics used to treat methicillin-susceptible *Staphylococcus aureus* (MSSA) bacteremia are cefazolin, nafcillin, and oxacillin. However, other β-lactams are used to treat MSSA bacteremia in certain clinical settings. Prior data suggest that the use of broad-spectrum β-lactams is associated with increased mortality compared to SOC antibiotics. Furthermore, broad-spectrum β-lactams may contribute to the development of antibiotic resistance and *Clostridioides difficile* infection (CDI). This study aimed to evaluate the outcomes of patients with MSSA bacteremia who received SOC versus broad-spectrum β-lactam antibiotics.

**Figure:**
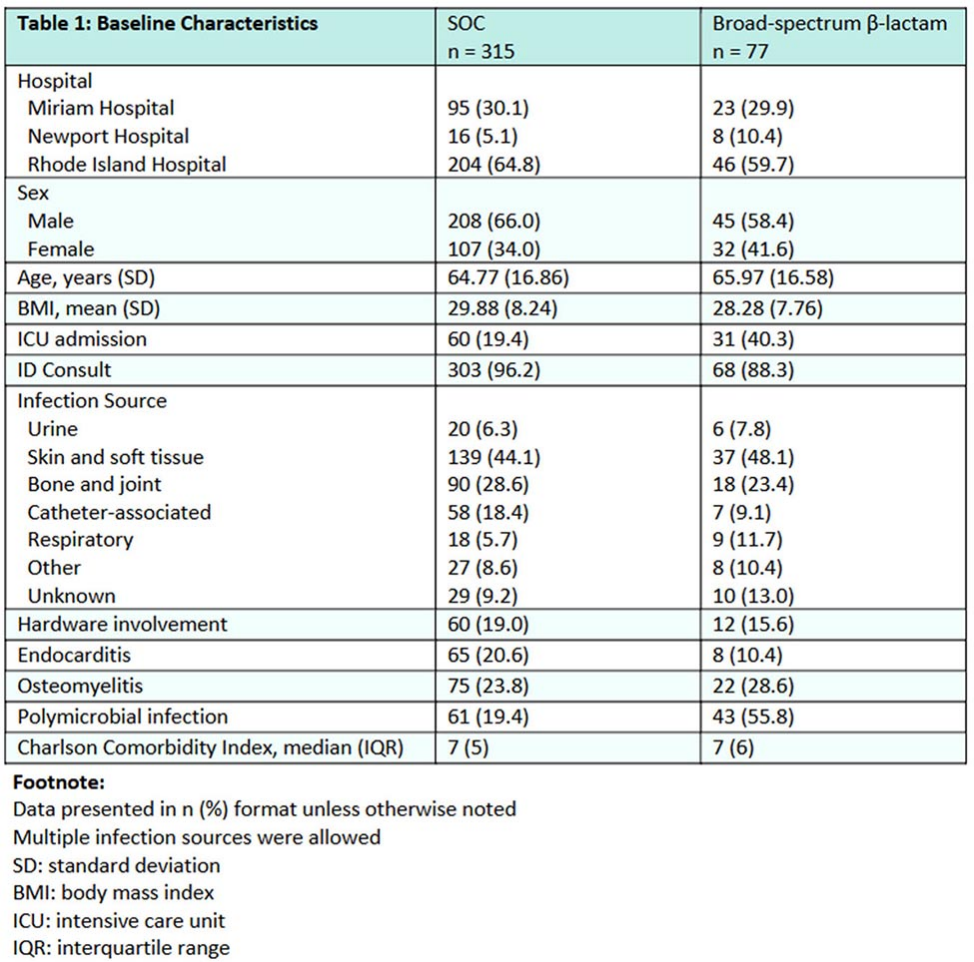

**Figure:**
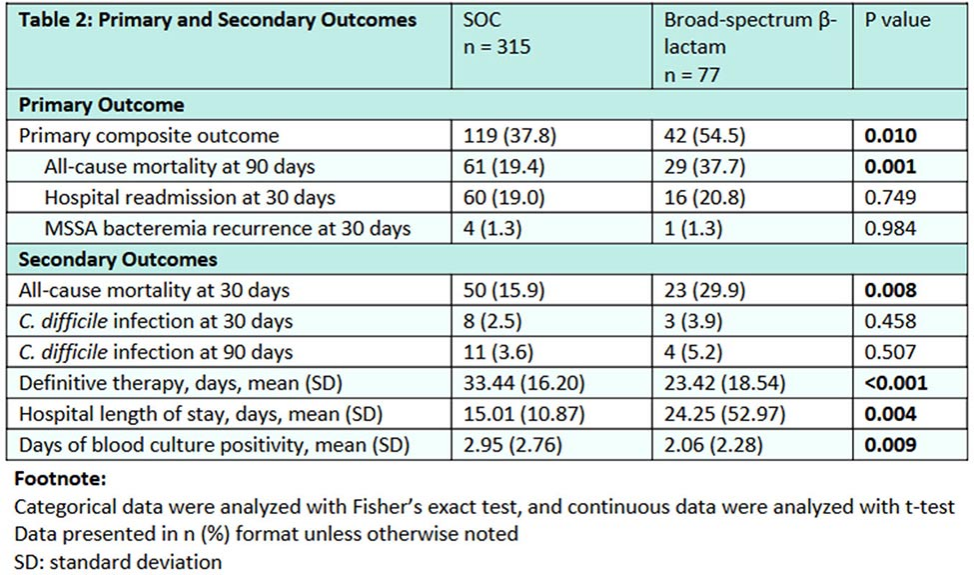

**Methods:**

Unique adult inpatients treated with β-lactam antibiotics for MSSA bacteremia between June 1st, 2021, and May 31st, 2024, were screened for inclusion. Patients were included in SOC (cefazolin, nafcillin) or broad-spectrum β-lactam (all other β-lactams) groups based on the definitive antibiotic, defined as the antibiotic received for the longest duration from 72 hours after index blood culture collection. The primary outcome was the composite of 90-day all-cause mortality from index blood culture collection, 30-day readmission from the end of therapy, and 30-day recurrence of MSSA bacteremia from the end of therapy. Secondary outcomes included CDI at 30 and 90 days from index blood culture collection, total duration of definitive therapy, length of hospital admission, and total days of blood culture positivity.

**Results:**

A total of 392 patients were included, 315 in the SOC group and 77 in the broad-spectrum β-lactam group. The primary outcome occurred in 161 patients, including 37.8% (119/315) of the SOC group and 54.5% (42/77) of the broad-spectrum β-lactam group (p = 0.010).

**Conclusion:**

The majority of adult inpatients in our health system from June 1st, 2021, to May 31st, 2024, with MSSA bacteremia were treated with SOC antibiotics. Despite infectious diseases (ID) consult directing therapy for the majority of patients, treatment of MSSA bacteremia with broad-spectrum β-lactams was significantly associated with worse clinical outcomes, driven by mortality. Further studies are needed to delineate risk factors for worse clinical outcomes with broad-spectrum β-lactams for MSSA bacteremia.

**Disclosures:**

All Authors: No reported disclosures

